# The Antifungal Eugenol Perturbs Dual Aromatic and Branched-Chain Amino Acid Permeases in the Cytoplasmic Membrane of Yeast

**DOI:** 10.1371/journal.pone.0076028

**Published:** 2013-10-18

**Authors:** Emad Darvishi, Mansoor Omidi, Ali Akbar Shahnejat Bushehri, Ashkan Golshani, Myron L. Smith

**Affiliations:** 1 Department of Agronomy and Plant Breeding, University of Tehran, Karaj, Iran; 2 Biology Department, Carleton University, Ottawa, Ontario, Canada; University of Wisconsin Medical School, United States of America

## Abstract

Eugenol is an aromatic component of clove oil that has therapeutic potential as an antifungal drug, although its mode of action and precise cellular target(s) remain ambiguous. To address this knowledge gap, a chemical-genetic profile analysis of eugenol was done using ∼4700 haploid *Saccharomyces cerevisiae* gene deletion mutants to reveal 21 deletion mutants with the greatest degree of susceptibility. Cellular roles of deleted genes in the most susceptible mutants indicate that the main targets for eugenol include pathways involved in biosynthesis and transport of aromatic and branched-chain amino acids. Follow-up analyses showed inhibitory effects of eugenol on amino acid permeases in the yeast cytoplasmic membrane. Furthermore, phenotypic suppression analysis revealed that eugenol interferes with two permeases, Tat1p and Gap1p, which are both involved in dual transport of aromatic and branched-chain amino acids through the yeast cytoplasmic membrane. Perturbation of cytoplasmic permeases represents a novel antifungal target and may explain previous observations that exposure to eugenol results in leakage of cell contents. Eugenol exposure may also contribute to amino acid starvation and thus holds promise as an anticancer therapeutic drug. Finally, this study provides further evidence of the usefulness of the yeast Gene Deletion Array approach in uncovering the mode of action of natural health products.

## Introduction

Infectious diseases are responsible for more than 17 million deaths each year worldwide [1]. Among other things, this is due to a lack of resources to combat diseases in impoverished regions and an increasing incidence of microbial resistance to existing antibiotics [2]. Phytomedicines are broadly accessible and could also provide a solution to the problem of drug resistance, as plant secondary metabolites may inhibit microbial growth by different mechanisms than the presently used antibiotics [3]. *Syzygium aromaticum* is a well-known aromatic plant species that is widely cultivated in Asian and African countries [4]. It is reported that the buds of *S. aromaticum* (cloves) are used in folk medicine as diuretic, odontalgic, stomachic, tonicardiac, and as a condiment with carminative and stimulant activity [5]. Clove essential oils have been described as having useful antiseptic and analgesic effects and are frequently used in dental medicine [6] and for sedating fish for research purposes. Several studies have also shown that clove oil has strong antibacterial, antifungal, antiviral and antioxidant activities. Many of these activities are believed to be due to the main biologically active phenolic component of clove essential oils, eugenol [6]–[10].

Despite many reports on the antimicrobial properties of the essential oils, including eugenol, found in most *Syzygium* species (e.g.s [6], [10]), the antifungal mode of action of these compounds was only investigated by *in vitro* pharmacological assays, and the precise cellular target of eugenol in fungi remains unclear. The leading view is that phenolic compounds such as eugenol disrupt the cytoplasmic membrane and result in cell leakage [10]–[12], and that a subsequent Ca^2+^ influx may serve a protective function against eugenol [13]. Considering that eugenol-rich essential oils are gaining increasing importance for their pronounced antimicrobial activity, the objective of our present research was to evaluate the antifungal activity and mechanism of action of eugenol using cell-based phenotypic screens in the yeast *Saccharomyces cerevisiae*.

## Materials and Methods

### Growth Media and Compounds

Standard rich (YPD) and synthetic complete (SC) media were used for the experiments [14]. *S. cerevisiae* cells were grown at 30°C for 1–2 days. The YPD medium containing Geneticin (G418; 200 µg/ml) was used for the maintenance of deletion strains carrying the G418^R^ selectable marker. G418 and eugenol were purchased from Sigma-Aldrich (Oakville, ON, Canada). Ethanolic root extracts of *Echinacea purpurea* were used as a positive control in membrane disruption assays and prepared as described previously [15].

### Antifungal Activity

*S. cerevisiae* (S288C) was used in antifungal activity assays. Minimum Inhibitory Concentration (MIC) for eugenol was measured using the broth microdilution assay protocol [16]. A three-fold serial dilution of eugenol (concentration range of 21.4 to 1.7×10^−7^ mg/ml) was added to sterile 96-well microtitre plates. Plates were incubated at 30°C for 1–2 days. Inhibition of growth was visually compared with control wells containing no eugenol.

### Gene Deletion Array (GDA) Analysis

For high throughput phenotypic screenings, approximately 4700 *MAT*a haploid gene deletion strains of *S. cerevisiae* derived from BY4741 (*MAT*a *ura3Δ0 leu2Δ0 his3Δ1 met15Δ0*) were maintained in an ordered array of approximately 384 individual strains in each of 16 plates [17]. YPD agar plates without (control), and with a subinhibitory concentration of eugenol (0.18 mg/ml, experimental), were inoculated by hand-pinning sets of 384 mutant strains per plate using a floating pin replicator as previously described [18]. After 1–2 days incubation at 30°C, digital images of the plates were captured and analyzed using Growth Detector software [19]. The relative size of colonies was used as a measure for growth differences under experimental and control conditions. Each experiment was repeated three times. Colonies that demonstrated 70% or more reduction in size in at least two replicates were classified as supersensitive (i.e. highly susceptible mutants). Deletion strains for genes associated with multidrug resistance were omitted from the list of supersensitives as they provide little information about the specific mode of activity of antifungal. Gene ontology annotation analysis was done with online software (gprofiler, http://biit.cs.ut.ee/gprofiler/; Profcom, http://webclu.bio.wzw.tum.de/profcom/; GeneMANIA, http://www.genemania.org/) and Saccharomyces Genome Database [20] was used for functional profiling of highly susceptible mutants in our large-scale experiment.

### Spot Test Analysis

Sensitivity of selected mutant strains identified in the GDA screens were confirmed by spot test analyses. Yeast cells were grown in YPD liquid medium to mid-log phase and 10-fold serially diluted. From each dilution, 15 µl was spotted on medium containing subinhibitory concentrations of eugenol (0.18 mg/ml), or without eugenol (control). The growth patterns were compared after 2 days at 30°C. Each experiment was repeated a minimum of three times.

### Liposome Leakage Assay

Carboxyfluorescein (CF, Life Technologies Inc., Burlington ON, Canada) was encapsulated in large unilamellar vesicles (LUVs) made of total yeast lipids (Avanti Polar Lipids, Alabaster AL, USA) in the quenched state for use in this experiment as described previously [21]. A black opaque microplate containing 190 µl of the optimal dilution of the LUVs suspension in each well had its fluorescent emissions measured with a FLUOstar microplate reader (OPTIMA BMG LABTECH Inc., Durham NC, USA) prior to addition of any of the test compounds to establish the fluorescence at time zero (F_0_). A row in the same plate was reserved for 190 µl per well of the HEPES buffer as a control to test for compound autofluorescence. Eugenol was two-fold serially diluted across a clear 96-well microplate in HEPES buffer and 10 µl from each well of the clear microplate was transferred to the well of a black opaque 96-well microplate. The final concentrations of the eugenol across the black opaque microplate were 1.6 to 0.003 mg/ml. Similarly, a dilution of *Echinacea purpurea* ethanol extract was used as a positive lysis control and the carrier solvent was used as the negative control. Following 1 h incubation at room temperature in the dark, the fluorescence units of each well was measured to determine F_t_. Immediately after, 20 µl of a 10% solution of Triton X-100 was added to each well causing 100% release and dequenching of CF. The microplate was read again after ∼10 min to determine the final fluorescence measurement F_100_ (100% fluorescence after addition of Triton X-100). The percentage of leakage for each point was calculated as: % Leakage = {(F_t_ – F_0_)/(F_100_ – F_0_)}×100.

### β-galactosidase Expression Assay

This assay used an inducible β-galactosidase reporter gene in p416 as described previously [22]. Briefly, cells of BY4741 harboring p416*GAL1*-*lac*Z were incubated in SC-ura medium containing 2% galactose with subinhibitory amounts (0.21 and 0.27 mg/ml) of eugenol and without eugenol (negative control). Cycloheximide (1 µg/ml, Sigma) was used as a positive control for translation inhibition. After 20 h at 30°C, yeast cells were harvested by centrifugation, cell density was measured at OD_600_ and β-galactosidase activity was measured as described in Miller *et al*. [23].

### Auxotroph Supplement Assay

All of the haploid gene deletion strains of *S. cerevisiae* derived from BY4741 (*MAT*a *ura3Δ0 leu2Δ0 his3Δ1 met15Δ0*) that were available in our GDA library and involved in tryptophan, phenylalanine, tyrosine or isoleucine biosynthesis pathways were grown in YPD liquid medium to mid-log phase and 10-fold serially diluted. From each dilution, 15 µl of each strain was spotted onto experimental (containing a subinhibitory 0.18 mg/ml concentration of eugenol) and control (no eugenol) plates of synthetic medium supplemented with four targeted amino acids (tryptophan, phenylalanine, tyrosine, isoleucine) together with uracil, leucine, histidine, methionine (that were essential for BY4741 background strain growth). The growth patterns were compared after 2 days at 30°C.

### Phenotypic Suppression Analysis

Overexpression constructs of four permeases that are either general (*GAP1*) or specific (*TAT1*, *TAT2* and *BAP2*) transporters for aromatic and branched-chain amino acids were obtained from the yeast gene overexpression array [24]. Suppression analysis was performed as described by Alamgir *et al*. [25]. Overexpression plasmids were transformed into the *aro1Δ* strain by the lithium acetate method [26]. Transformants were grown overnight in SC-ura medium, adjusted to OD_600_ ∼1.0 and diluted 1∶300 before 100 µl aliquots of the diluted broth cultures were added to each well of a sterile microtiter plate. Sensitivity to 0.18 mg/ml eugenol by the transformants containing the overexpression constructs was compared to that with a control plasmid after 2 days growth at 30°C by measuring the optical density (OD_600_) of cells in each well with a FLUOstar microplate reader (OPTIMA BMG LABTECH Inc., Durham NC, USA).

### Statistical Analyses

Statistical significance in the data sets was assessed by Student T-test using Microsoft Excel 2007 (Microsoft Corporation, USA). The difference was considered to be statistically significant when *P*-value≤0.05.

## Results and Discussion

### Antifungal Activity of Eugenol

We determined that the minimum inhibitory concentration (MIC_100_) for eugenol was in the range of 0.27–0.32 mg/ml. This MIC_100_ was defined as the lowest concentration that resulted in complete inhibition of visible growth of *S. cerevisiae* strain S288C after 2 days using a broth microdilution assay [16].

### GDA shows Eugenol Interacts with Aromatic and Branched-chain Amino Acid Pathway(s)

**Figure 1:**
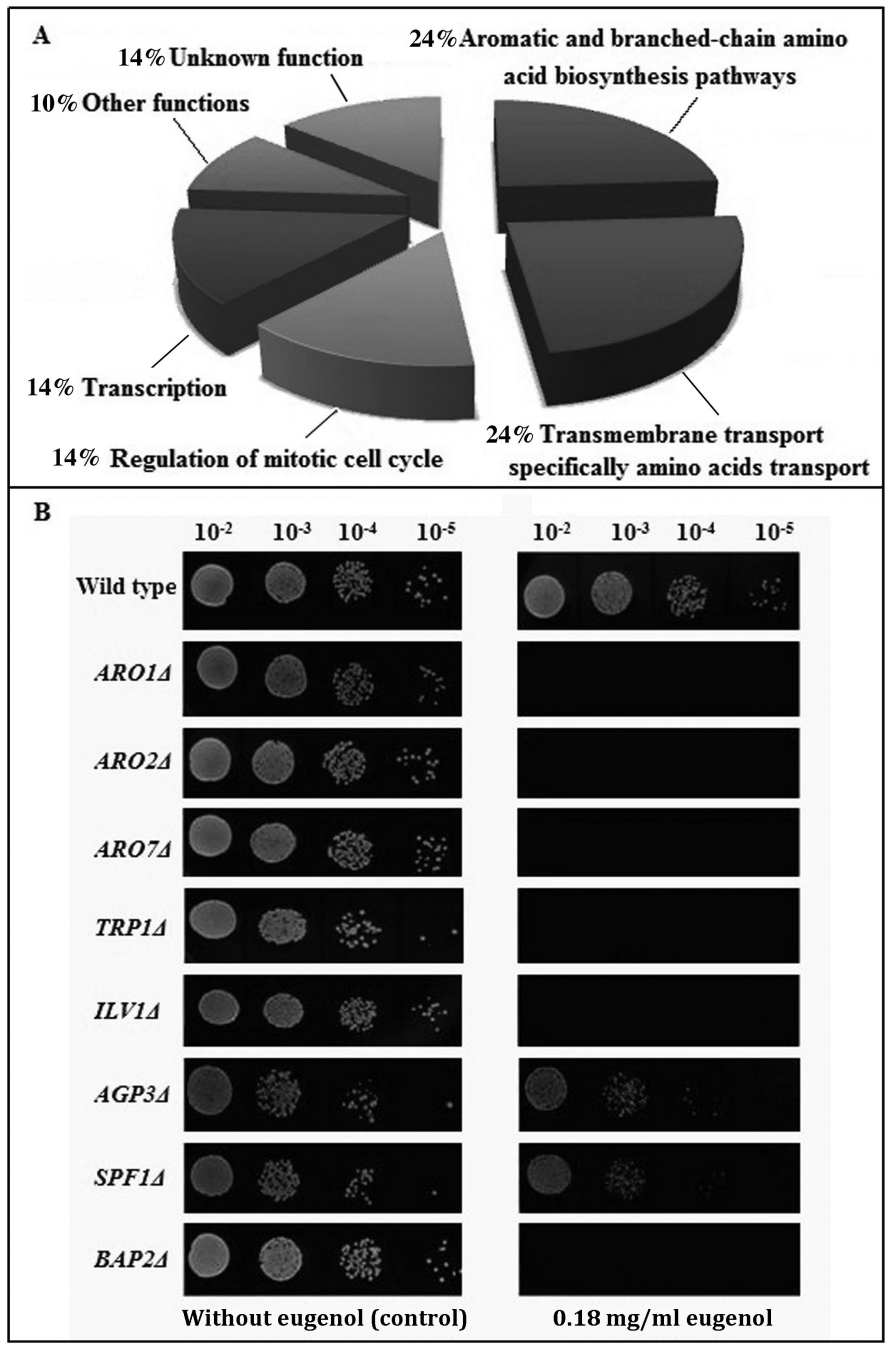
Nearly half of eugenol-sensitive strains have deletions in genes involved in aromatic and branched-chain amino acid synthesis or uptake. (A) The haploid non-essential yeast gene deletion array was subjected to a subinhibitory concentration of eugenol. Colony size reduction was used to detect sensitivity. The mutants most sensitive to eugenol were clustered according to the cellular processes in which their deleted genes participated. (B) Eugenol-sensitive strains identified by GDA were verified by drop out plates. Wild type and eight randomly selected gene deletion mutant strains that were eugenol-sensitive based on GDA analysis were 10-fold serially diluted and spotted on solid YPD medium with a subinhibitory concentration (0.18 mg/ml) of eugenol or without eugenol (control). The plates were incubated at 30°C for 1–2 days and then photographed. All deletion mutants selected exhibited increased sensitivity to eugenol, providing verification of the GDA analysis.

A range of subinhibitory concentrations (0.16–0.21 mg/ml) was initially tested using a representative plate of yeast deletion strains to determine that a concentration of 0.18 mg/ml gave approximately 1% supersensitives. The entire haploid yeast gene deletion array (yGDA) was then replica plated onto medium with this subinhibitory concentration of eugenol and subsequent colony size measurements revealed that 21 deletion mutants were super-sensitive to eugenol (Table 1). The deleted genes are not normally required for growth under laboratory conditions, and slow growth by these supersensitive deletion strains is likely a result of a chemical-genetic interaction. The genes deleted in supersensitive strains were clustered based on the cellular processes in which they participate (Figure 1A). Of the 21 most sensitive gene deletions, 48% have known roles in aromatic and branched-chain amino acid biosynthesis and transport pathways (*P*-value: 8.6×10^−5^). These genes include *ARO1*, *ARO2*, *ARO7*, *TRP1*, *ILV1* which are involved in aromatic and branched-chain amino acid biosynthesis pathways (24%) and *BAP2*, *AGP3*, *SLM4*, *SPF1*, *WSC4* which are linked to transmembrane transport and/or linked to amino acid transport (24%) through the cytoplasmic membrane. Specifically, BAP2 and AGP3 encode amino acid permeases [27], [28]. Slm4p is a component of the GSE complex that is required for proper sorting of amino acid permease Gap1p from the late endosome to the plasma membrane [29]. Spf1p, a P-type ATPase that may provide an electrochemical gradient across the plasma membrane needed for active amino acid transport, has physical interactions with amino acid permeases such as Bap2p and Gap1p, as well as Dip5p and Can1p that are dicarboxylic amino acid and arginine permeases, respectively [30]. Finally, Wsc4p interacts genetically with amino acid transporter, Tat1p, and is both co-expressed and co-localized with Gap1p [31].

**Table 1:** List of eugenol-sensitive gene deletion mutants from GDA analysis that showed greater than 70% reduction in colony size.

Gene function	Systematic Name	Standard Name	Average % colony size reduction
Aromatic and branched-chain amino acid biosynthesis pathways			
	*YDR127W*	*ARO1*	95.1
	*YGL148W*	*ARO2*	97.6
	*YPR060C*	*ARO7*	91.6
	*YDR007W*	*TRP1*	93.1
	*YER086W*	*ILV1*	96.8
Transmembrane transport specifically amino acids transport			
	*YBR068C*	*BAP2*	94.3
	*YFL055W*	*AGP3*	71.2
	*YBR077C*	*SLM4*	90.7
	*YEL031W*	*SPF1*	70.7
	*YHL028W*	*WSC4*	77.1
Regulation of mitotic cell cycle			
	*YOR026W*	*BUB3*	86.3
	*YNL273W*	*TOF1*	75.5
	*YGL173C*	*KEM1*	84.8
Transcription			
	*YBR245C*	*ISW1*	91.6
	*YHL025W*	*SNF6*	76.1
	*YPL042C*	*SSN3*	72.0
Other functions			
	*YDR312W*	*SSF2*	82.1
	*YMR202W*	*ERG2*	95.3
Unknown function			
	*YDR442W*	*YDR442W*	88.5
	*YMR326C*	*YMR326C*	78.9
	*YPR087W*	*VPS69*	78.2

Among the next largest clusters, approximately 14%, 14% and 10% of sensitive strains are associated with regulation of mitotic cell cycle, transcription, and other functions, respectively (excluding genes with unknown functions). These smaller clusters in the profile could represent additional target sites (side effects) of eugenol on yeast cells.

### Spot Test Analysis Verifies GDA

To investigate the accuracy of our large-scale approach for identifying eugenol-sensitive mutants, eight deletion strains that were supersensitive to eugenol based on the GDA assay were randomly selected and subjected to spot test analyses (Figure 1B). These spot test assays confirmed that deletion of these genes confers increased sensitivity to eugenol, and verified the large-scale screen based on the GDA approach.

### Hypotheses on the Antifungal Mode of Action of Eugenol

**Figure 2:**
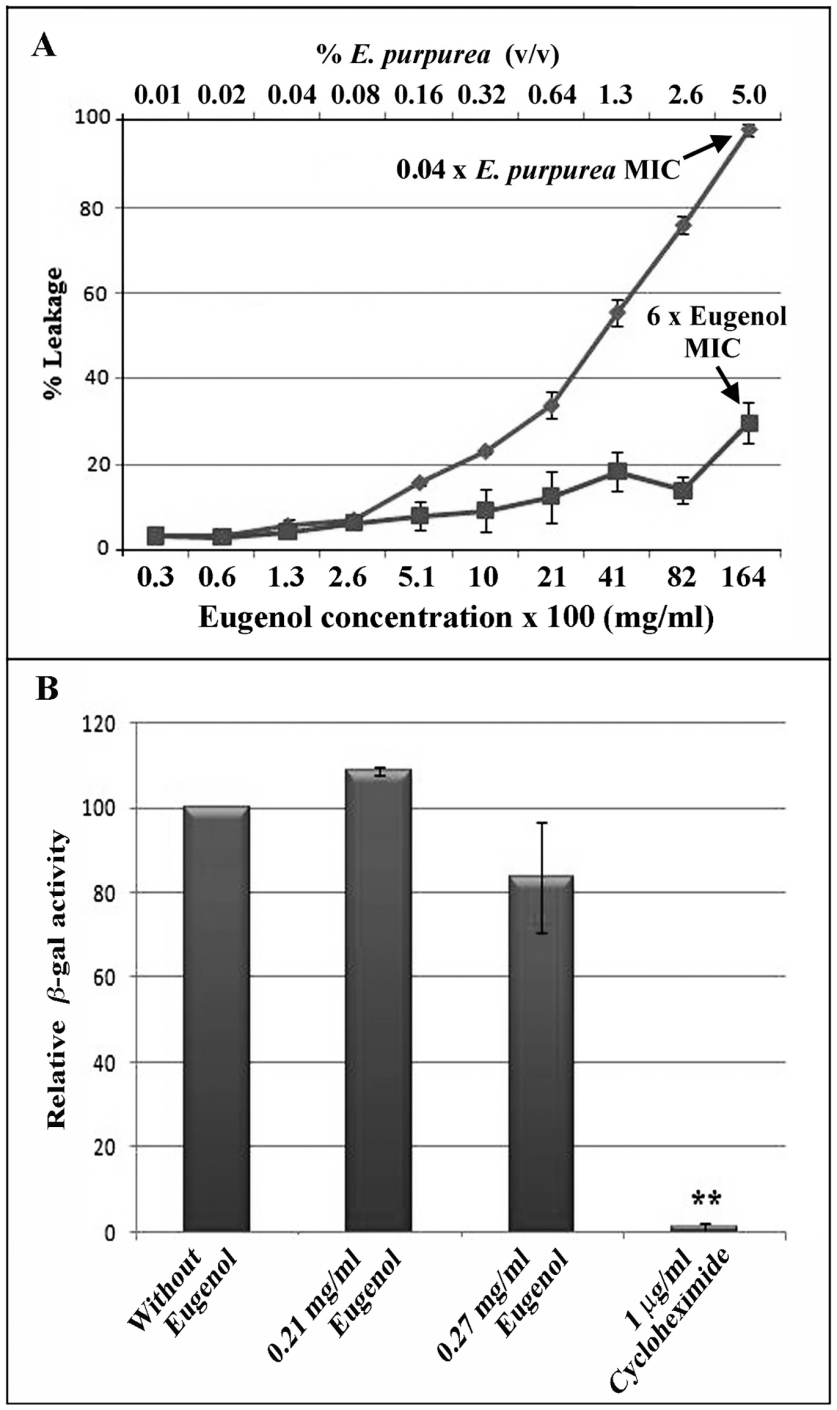
Eugenol does not induce leakage of liposomes made of total yeast lipids or inhibit protein synthesis. (A) Release of carboxyfluorescein from large unilamellar vesicles (LUVs) over a series of eugenol concentrations (0.003 to 1.6 mg/ml, bottom axis) was compared to 100% release from liposome exposed to Triton-X 100. *Echinacea purpurea* extract was used as a positive lysis control over a concentration gradient of 0.01 to 5% (top axis). Data correspond to the mean % leakage values (±SD) of three independent experiments. (B) Yeast exposed to subinhibitory (0.21 mg/ml) or inhibitory (0.27 mg/ml) concentrations of eugenol do not have significantly decreased β-galactosidase activity in comparison to the untreated control. In contrast, the inhibitor of protein translation, cycloheximide, significantly reduced β-galactosidase activity in the assay. These observations indicate that eugenol does not reduce efficiency of translation in yeast as would be expected for compounds that perturb the intracellular pool of amino acids. The values are expressed as mean ±SD of triplicates, difference between treatment and untreated control are indicated as p<0.05 (*) and p<0.01 (**).

Existing reports on the antimicrobial activity propose that phenolic compounds, such as eugenol and its analogs, compromise the structural and functional integrity of the cytoplasmic membrane and thus cause cell leakage [10]–[12]. To test this possibility, we examined the effect of eugenol on a model of the *S. cerevisiae* cytoplasmic membrane by monitoring the release of carboxyfluorescein (CF) from large unilamellar vesicles (LUVs). As seen in Figure 2A, only ∼12.5% leakage occurred at the eugenol MIC_100_ (0.27 mg/ml), and liposomes were only slightly destabilized even at concentrations of 1.6 mg/ml (6 times the eugenol MIC_100_). In contrast, *Echinacea purpurea* extract (positive control) caused 100% leakage at a concentration that corresponds to 0.04 times the yeast MIC_100_ value. This indicates that eugenol’s antifungal mode of action is not likely related to membrane disruption *per se* as suggested previously [11].

According to our GDA analysis, we can hypothesize two additional antifungal modes of action: (i) Eugenol may interfere with factors that are involved in aromatic and branched-chain amino acid biosynthesis pathways, and thus is expected to reduce the internal pool of these amino acids and, as a consequence, efficiency of translation in yeast cells. (ii) Eugenol may interfere with transporters, particularly aromatic and branched-chain amino acids permeases, in the cytoplasmic membrane of yeast cells. We therefore designed secondary assays to test these two hypotheses.

### Eugenol does not Significantly Reduce Translation Efficiency

According to the first hypothesis, the efficiency of translation in yeast cells would decrease in the presence of eugenol relative to the untreated control. To investigate this possibility, we used an inducible β-galactosidase reporter construct and found that the addition of subinhibitory (0.21 mg/ml) or inhibitory (0.27 mg/ml) concentrations of eugenol to yeast cells did not significantly decrease β-galactosidase activity in comparison to the untreated control (*P≥*0.1, Figure 2B). In contrast, 1 µg/ml of cycloheximide, a known inhibitor of protein translation, significantly reduced β-galactosidase activity in the assay. These observations do not support the first hypothesis, that eugenol interferes with factors involved in aromatic and branched-chain amino acid biosynthesis, since any perturbation by eugenol of the amino acid pool would likely reduce the efficiency of translation in yeast.

### Eugenol Blocks Uptake of Aromatic and Branched-chain Amino Acids

**Figure 3:**
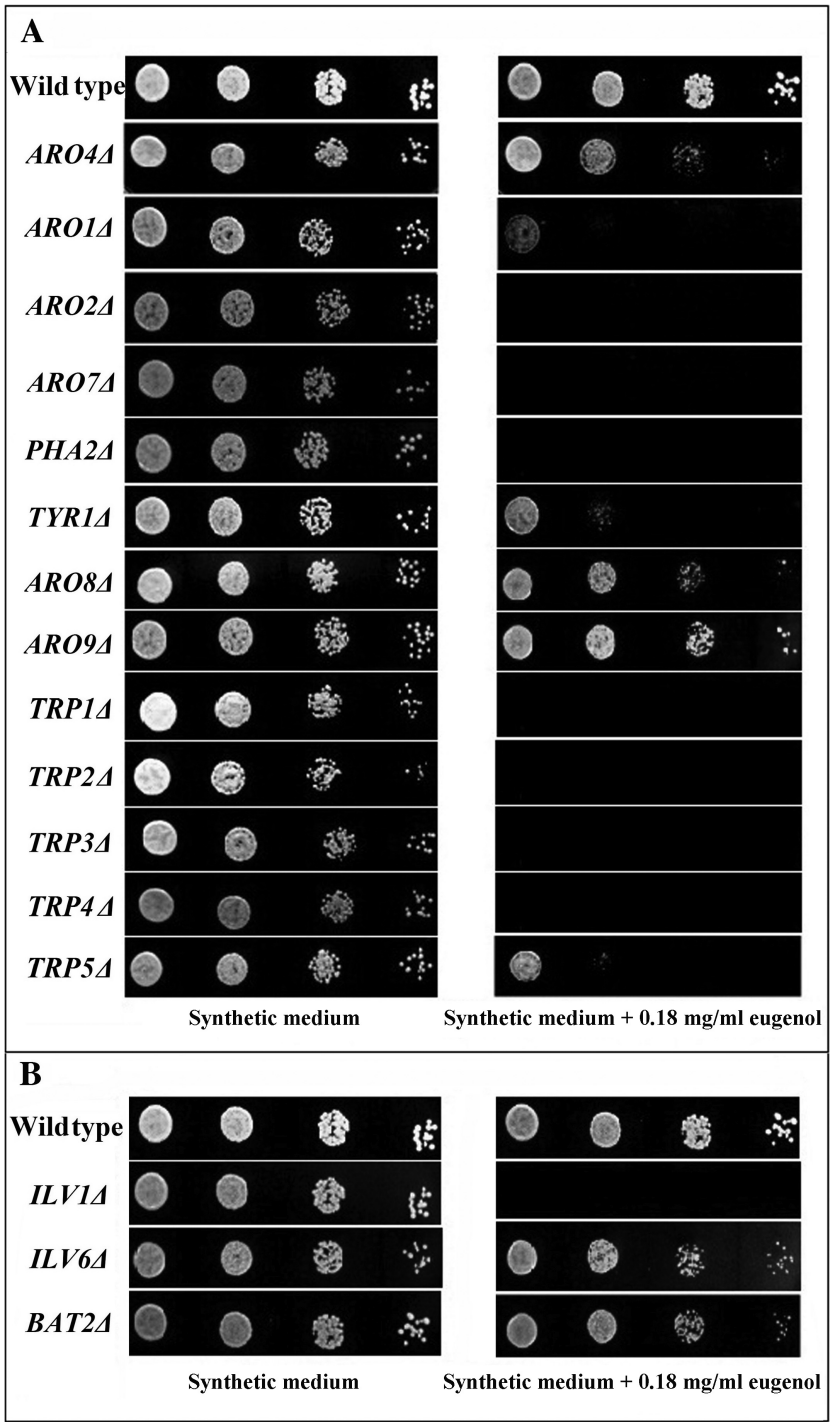
Auxotroph supplement assay shows that eugenol inhibits the functions of aromatic and branched-chain amino acid transporters. Strains of *S. cerevisiae* from GDA library were selected with gene deletions in the tryptophan, phenylalanine, tyrosine (A) or isoleucine (B) biosynthesis pathways. Cultures were 10-fold serially diluted and spotted on synthetic medium supplemented with tryptophan, phenylalanine, tyrosine and isoleucine, either containing a subinhibitory concentration of eugenol (0.18 mg/ml) or without eugenol (control). The plates were incubated at 30°C for 1–2 days and then photographed.

To test our second hypothesis, that eugenol interferes with specific amino acid transporters, we used an auxotroph supplement assay. The basis of this assay is as follows. A yeast strain that carries a mutation in a gene encoding an enzyme in a tryptophan, phenylalanine, tyrosine and/or isoleucine biosynthesis pathway will only be able to grow if these amino acids are provided in the culture medium. In contrast, auxotrophic strains are expected to grow poorly on synthetic medium supplemented with these amino acids if eugenol is present and interferes with aromatic and branched-chain amino acids permeases. Using the auxotroph supplement assay we examined the 13 strains shown in Figure 3A that carry mutations in aromatic amino acid biosynthesis genes. Of these 13 strains, only three (*aro4Δ, aro8Δ* and *aro9Δ*) grew well on a synthetic medium supplemented with aromatic amino acids that contained a subinhibitory concentration of eugenol (0.18 mg/ml). The remaining ten strains grew well on aromatic amino acid-supplemented medium, but grew poorly or not at all when eugenol was included in the medium. It has been reported that neither of the *ARO8* or *ARO9* single mutants display any nutritional requirements on minimal ammonia medium whereas the *ARO8* and *ARO9* double mutant is auxotrophic for both phenylalanine and tyrosine [32]. Similarly, there is no evidence that the *ARO4* single mutant displays an auxotrophic phenotype for aromatic amino acids [20]. In addition, as seen in Figure 3B, among the three strains with mutations in the branched-chain amino acid (isoleucine) biosynthetic pathway (*ilv1Δ*, *ilv6Δ* and *bat2Δ* were available in our GDA library), only the *ilv1Δ* strain could not grow on synthetic medium supplemented with isoleucine in the presence of 0.18 mg/ml eugenol. Kispal *et al*. [33] reported that on glucose-containing media, a single deletion of either of the two *BAT* genes (*BAT1* or *BAT2*) does not impair cell growth, but deletion of both genes results in branched-chain amino acid auxotrophy and severe growth retardation. In addition, there is no evidence that mutations in the *ILV6* gene results in an auxotrophic phenotype for isoleucine [20]. These reports are consistent with our results and noticeably confirm our second hypothesis that eugenol interferes with transporters responsible for uptake of aromatic and branched-chain amino acids across the yeast cytoplasmic membrane. This putative interference with amino acid uptake, in itself, would not appear to fully explain the antifungal mode of action of eugenol since growth by amino acid prototrophs is nevertheless inhibited by eugenol.

### Phenotypic Suppression Assay

**Figure 4:**
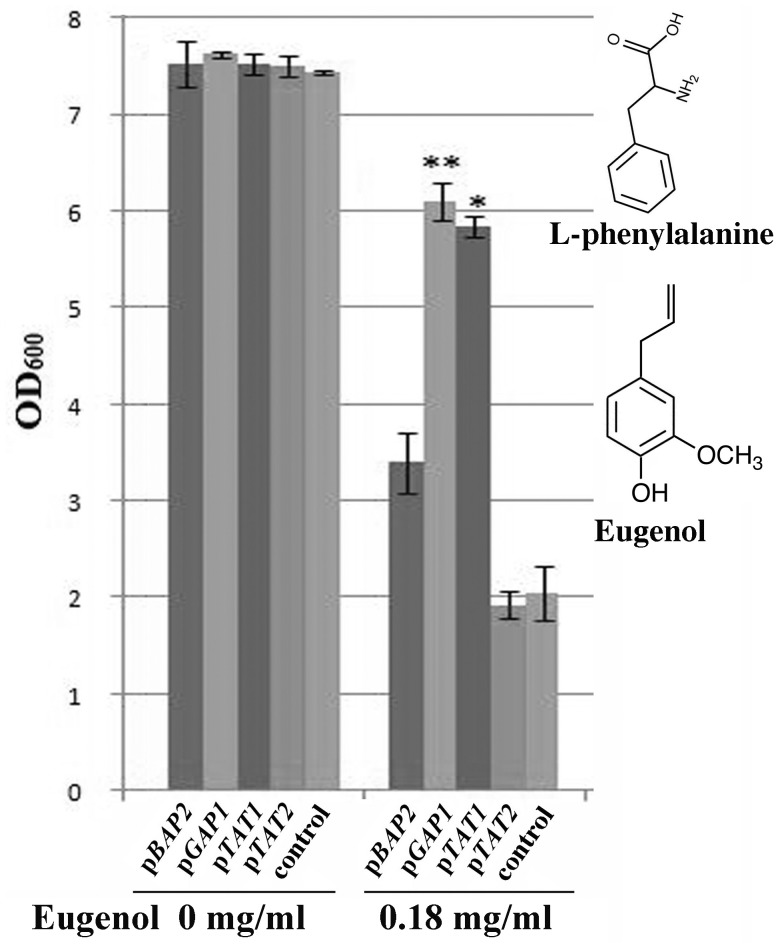
Phenotypic suppression assay shows that Tat1p and Gap1p permeases are targets of eugenol in the yeast cytoplasmic membrane. Overexpression constructs of four permeases, *BAP2, GAP1*, *TAT1* and *TAT2* were transformed separately into the *aro1Δ* strain. Transformants were grown overnight in SC-ura medium, diluted 1∶300 and then added to each well of a sterile microtiter plate with or without 0.18 mg/ml eugenol. Growth of the *aro1Δ* transformants containing the overexpression constructs were compared to one with a control plasmid by measuring the optical density of cells in each well (OD_600_). The values are expressed as mean (±SD, n = 3) and significant differences between treatment and plasmid control are indicated as *P<0.05* (*) and *P<0.01* (**) based on Student T-test. Inset: eugenol is structurally similar to aromatic amino acids and is synthesized in plants via the phenylpropanoid pathway from phenylalanine.

To further test whether eugenol interferes with aromatic and branched-chain amino acid permeases, we expressed *BAP2*, *GAP1*, *TAT1* and *TAT2* (which encode general or specific transporters for aromatic and branched-chain amino acids in the cytoplasmic membrane of yeast) from high copy number plasmids in the *aro1Δ* strain. As indicated in Figure 4, the growth defect of the *aro1Δ* strain grown in SC-ura medium containing 0.18 mg/ml eugenol was partially compensated by the overexpression of *GAP1* (*P<*0.01) and *TAT1* (*P<*0.05) in comparison to the *aro1Δ* strain with a control plasmid. However, no significant differences in growth were evident when the *aro1Δ* strain carried either *BAP2* or *TAT2* overexpression constructs compared to that with the control plasmid (*P*>0.1). Therefore, we propose that eugenol specifically interferes with permeases with a dual transport function for both aromatic and branched-chain amino acids (i.e. Tat1p and Gap1p), rather than Bap2p and Tat2p which are high-affinity transporters for branched-chain and aromatic amino acids, respectively [20], [34], [35]. Furthermore, the inferred eugenol-specific interaction with Tat1p rather than Tat2p provides additional evidence for substrate specificity of these two permeases as reported by Regenberg *et al*. [28].

Eugenol is a member of the phenylpropanoid class of plant secondary metabolites. Aromatic amino acids, specifically phenylalanine, are precursors of eugenol in the phenylpropanoid biosynthesis pathway [36]. As shown in the inset of Figure 4, the molecular structure of eugenol is very similar to these aromatic amino acid precursors and because of these structural similarities eugenol may interfere with active sites of both Tat1p and Gap1p permeases. While our results indicate that eugenol does not likely perturb the phospholipid bilayer directly, binding of eugenol to these membrane-bound permeases could alter their permeability or cause conformational changes in the targeted permeases that disrupt the yeast cytoplasmic membrane to account for release of intracellular components as reported previously [10]–[12]. Indeed, current models recognize that there is a tight association between proteins, including Gap1p in yeast, and lipids in the cell membrane [37]. However, this model seems incongruent with the partial rescue of *aro1Δ* strain from eugenol sensitivity that is observed when Gap1p and Tat1p are overexpressed; in this case, an increased abundance of eugenol targets may be expected to increase cytoplasmic leakage and thus sensitivity to eugenol. Further investigations should be carried out to test whether or not there is a direct effect by eugenol on the conformation of Tat1p and Gap1p permeases. Another possible explanation is that Gap1p and Tat1p serve other important functions in the cell. For example, in addition to amino acid transport, Gap1p plays a role in amino acid sensing in a protein kinase A (PKA)-mediated protein phosphorylation cascade [38]. Perturbation of these additional functions may result in cell death and subsequent cytoplasmic leakage.

Approximately 45% of the genes in yeast are homologous to mammalian genes (BLAST e-value<10^−10^) [39], supporting the view that chemical-genetic profiles obtained from yeast can reflect disease processes in human cells [40]. It should be noted in this context, that amino acid starvation is an effective strategy for cancer therapy. It has been demonstrated that murine and human melanoma cells are induced to undergo apoptosis by phenylalanine and tyrosine starvation [41]. Tsukahara *et al*. showed that the novel anticancer chemical E7070 inhibits leucine and uracil transporters in the fission yeast, *Schizosaccharomyces pombe*, and may also target mammalian transporters [42]. It has also been reported that inhibitors of the mammalian amino acid permease, LAT1, that preferentially transports branched-chain and aromatic amino acids through the plasma membrane, may be an effective therapeutic option for human astrocytic tumors [43]. Based on these considerations, amino acid permease inhibitors such as eugenol may hold chemotherapeutic promise for human cancers. However, detailed studies are required to profile the genome-wide effects of eugenol in human cell lines to exploit its therapeutic potential more effectively.

The interesting mode of action of eugenol identified herein for the first time is notable as being distinct from those of commercially available antifungals such as azoles and amphotericin B. Of concern, cross-resistance to amphotericin B and azole antifungals can result from a single mutation in genes involved in ergosterol biosynthesis [44]. Given their different targets in the cell, permeases vs ergosterol, eugenol would likely be useful against increasingly common clinical isolates of pathogenic fungi that are cross-resistant to amphotericin B and azole drugs.
